# Species-wide genome sequence and nucleotide polymorphisms from the model allopolyploid plant *Brassica napus*

**DOI:** 10.1038/sdata.2015.72

**Published:** 2015-12-08

**Authors:** Thomas Schmutzer, Birgit Samans, Emmanuelle Dyrszka, Chris Ulpinnis, Stephan Weise, Doreen Stengel, Christian Colmsee, Denis Lespinasse, Zeljko Micic, Stefan Abel, Peter Duchscherer, Frank Breuer, Amine Abbadi, Gunhild Leckband, Rod Snowdon, Uwe Scholz

**Affiliations:** 1 Leibniz Institute of Plant Genetics and Crop Plant Research (IPK) Gatersleben, Corrensstraße 3, Stadt Seeland 06466, Germany; 2 Justus Liebig University, Department of Plant Breeding, Heinrich-Buff-Ring 26-32, Gießen 35392, Germany; 3 Syngenta France SAS, 12 chemin de l’Hobit, Saint-Sauveur 31790, France; 4 Deutsche Saatveredelung AG, Weissenburger Straße 5, Lippstadt 59557, Germany; 5 Limagrain GmbH, Salder Str. 4, Peine 31226, Germany; 6 Bayer Crop Science AG, Streichmühler Str. 8, Grundhof 24977, Germany; 7 KWS Saat AG, Grimsehlstr. 31, Einbeck 37555, Germany; 8 NPZ Innovation GmbH, Hohenlieth-Hof, Holtsee 24363, Germany; 9 German Seed Alliance GmbH, Neue Schönholzer Str. 12, Berlin 13187, Germany

**Keywords:** Next-generation sequencing, DNA sequencing, Plant genetics, Agricultural genetics, Plant breeding

## Abstract

*Brassica napus* (oilseed rape, canola) is one of the world’s most important sources of vegetable oil for human nutrition and biofuel, and also a model species for studies investigating the evolutionary consequences of polyploidisation. Strong bottlenecks during its recent origin from interspecific hybridisation, and subsequently through intensive artificial selection, have severely depleted the genetic diversity available for breeding. On the other hand, high-throughput genome profiling technologies today provide unprecedented scope to identify, characterise and utilise genetic diversity in primary and secondary crop gene pools. Such methods also enable implementation of genomic selection strategies to accelerate breeding progress. The key prerequisite is availability of high-quality sequence data and identification of high-quality, genome-wide sequence polymorphisms representing relevant gene pools. We present comprehensive genome resequencing data from a panel of 52 highly diverse natural and synthetic *B. napus* accessions, along with a stringently selected panel of 4.3 million high-confidence, genome-wide SNPs. The data is of great interest for genomics-assisted breeding and for evolutionary studies on the origins and consequences in allopolyploidisation in plants.

## Background and Summary

Detailed knowledge of genome-level diversity is an important prerequisite for understanding the inheritance of complex traits related to crop performance, and for implementation of genome-based performance prediction to accelerate breeding progress. Ultra-high throughput, array-based genotyping platforms enable cheap and efficient whole-genome profiling in any species for which suitable DNA sequence polymorphism datasets are available. The recent publications of reference sequences for the allopolyploid genome of oilseed rape^1^ and its diploid progenitor species, *Brassica rapa*^2^ and *Brassica oleracea*^3^, open the possibility for large-scale genomic resequencing to discover and utilise DNA sequence diversity in the primary and secondary gene pools of these important crops.

Furthermore, the unique ability to generate synthetic *B. napus*, from *de novo* interspecific hybridisation between *B. rapa* and *B. oleracea*, provides the opportunity to capture completely novel *B. napus* variants to diversify the primary gene pool of cultivated oilseed forms^4^. Synthetic *B. napus* has also become an important model to study homoeologous chromosome pairing, inter-subgenomic chromosome exchanges and gene conversions resulting from *de novo* allopolyploidisation. Numerous examples demonstrate the influence and consequences of natural or artificial selection on particular chromosome exchanges for the expression of important agronomic traits^1,5^. To capture, recombine and utilise such variation for high-resolution trait dissection and genomics-based pre-breeding, we first established a highly diverse panel of 50 natural and synthetic *B. napus* accessions. These were subsequently used as founder lines for the development of large populations for nested association mapping (NAM^6^) and heterotic haplotype capture (HHC^4^). 30 of the founder accessions were selected to sample allelic variation as evenly as possible across the gene pools of diverse winter-type *B. napus* morphotypes, including fodder rapes, kales, and old European and Asian oilseed forms (*B. napus* ssp. *napus*), along with rutabaga/swede (*B. napus* ssp. *napobrassica*). The remaining 20 founder accessions comprise synthetic *B. napus*, generated through interspecific hybrids of genetically highly divergent representatives of the diploid A and C donor genomes of the allopolyploid *B. napus*. Two additional, modern breeding lines were used as recurrent parents for the generation of the NAM lines and HHC populations, respectively^4^.

Whole-genome resequencing data was generated for the 50 accessions from the diversity panel, and for the two recurrent parents, using the Illumina HiSeq 2000 next-generation sequencing platform. Paired-end, 100 bp sequence reads were generated to an average depth of between 12x and 15x genome coverage. A total of 3.4 billion quality-trimmed paired reads (685 Gb) was utilized for the alignment to the *B. napus* Darmor-*bzh* v4.2 reference genome. Using different, stringent variant calling strategies, a total of 4.3 million high-confidence, genome-wide single-nucleotide polymorphisms (SNPs) were detected across the 52 genomes.

This paper reports these DNA sequence and SNP variant datasets in their entirety. These data are expected to be of considerable interest to breeders and geneticists working with *Brassica* crops, and to evolutionary biologists investigating polyploid evolution and the genome-scale consequences of natural or artificial selection. Furthermore, the data represent a foundation for development of new, ultrahigh-density variant screening arrays for population-level trait analysis and genomic selection.

## Methods

### The Pre-BreedYield diversity set (PBY collection)

The primary sequencing panel of 30 natural *B. napus* accessions was selected from a species-wide pool of more than 500 highly diverse *B. napus* accessions described by^7^. By analysing the sequences of conserved genes across this entire diversity set we selected 30 homozygous inbred accessions that best represented the species-wide diversity present in this panel. The selected accessions include fodder rapes, kales, old European and Asian oilseed forms (all *B. napus* ssp. *napus*), along with rutabaga/swede forms (*B. napus* ssp. *napobrassica*). The genetic diversity of the 30 natural *B. napus* accessions was further expanded by adding 20 synthetic *B. napus* accessions developed by embryo rescue from interspecific hybridizations between highly diverse parental origins (Table 1). The development and origins of the synthetic *B. napus* accessions are described in detail by Girke^8^ and Jesske^9^. The Pre-BreedYield (PBY) collection was completed by two additional, modern breeding lines that were used as recurrent parents for the generation of the NAM lines and HHC populations, respectively^4^. Whole-genome shotgun sequencing (WGS) was performed for each of the 52 lines included in the PBY collection (Table 2 (available online only)).

### Sample preparation and whole-genome sequencing

The 52 *B. napus* accessions, including 30 adapted, 20 resynthesized and 2 elite lines, were resequenced using an Illumina HiSeq 2000 platform. For each sample 1 ug of gDNA has been fragmented into small fragments of 400 bp using the Covaris standard technology without any genomic reduction. The NEBNext DNA library kit was used to prepare the library, following the standard protocol. Cleaning and size selection were done using Ampure magnetic beads. Each amplified fragment has been paired end sequenced, generating short-reads of 100 bp to an average coverage of 12–15x (corresponding to 20 Gb per accession). The full sequence data for all 52 accessions is archived at the European Nucleotide Archive (www.ebi.ac.uk/ena) under the project numbers PRJEB5974 (Data Citation 1) and PRJEB6069 (Data Citation 2).

### Raw data processing

The quality of the raw sequence data was validated using the *FastQC* software (http://www.bioinformatics.babraham.ac.uk/projects/fastqc/). Adapters were removed using *cutadapt* (https://cutadapt.readthedocs.org/) and the reads were trimmed for low quality bases with the *fastx* quality trimmer algorithm (http://hannonlab.cshl.edu/fastx_toolkit/), using the settings −t 30 −l 90 for trimming bases with a Phred quality score below 30 and removing reads shorter than 90 nt. Paired reads were then synchronized using an in-house Perl script (Data Citation 3). General quality statistics of raw and trimmed reads were generated using *fastx_quality_stats* (http://hannonlab.cshl.edu/fastx_toolkit/). In total over 3.4 billion quality-trimmed, paired-end reads were used, providing a total pre-processed data volume of 685 Gbp. Average G/C content changed slightly from 36.34 to 35.35 percent after trimming of raw reads, whereby the diverse genotype collection showed a range of G/C content from 34.87 to 36.33%, emphasizing the high variability. These values are comparable to previous calculations of 35.4% in *B. rapa* and 36.0% in *B. oleracea*^10^. In consequence of the stringent quality trimming the average sequencing depth per genotype decreased to ~11.7 fold, with a Q1 quartile of ~10.0 fold and a Q3 quartile of ~12.6 fold. This provides a sufficient coverage for in-depth diversity study across all genotypes. Table 2 (available online only) gives a complete list of basic statistics describing the raw and trimmed sequence data.

### Whole genome alignment against the *Brassica napus* Darmor-*bzh* reference genome

The 1,130 Mb genome of the allopolyploid species *B. napus*^1^ is characterized by two highly similar sub-genomes. The A subgenome, derived from *B. rapa*, consists of 10 chromosomes, while the C subgenome contributed by *B. oleracea* has 9 chromosomes. The preprocessed sequence reads were mapped to the *B. napus* Darmor-*bzh* v4.2 reference genome using the *SOAP2* software^11^ with the options −m 0, −x 500, −v 2. To achieve high confidence when aligning reads to the allopolyploid genome, we used an un-gapped alignment. This approach considers the close similarity of the two subgenomes, increasing the ratio of reads that were aligned to the correct subgenome. The *SOAP2* output files with the positions of the aligned reads were converted to the Sequence Alignment/Map (SAM) format applying the 'soap2sam.pl' script (http://soap.genomics.org.cn/down/soap2sam.tar.gz), and then further converted into the binary version of the SAM format (BAM) using *SAMtools* view command^12^.

For the 52 different accessions an average of ~90 million reads per accession were aligned as proper read pairs, ranging from ~66.0 million reads at the first quartile (Q1) to ~102.6 million reads at the third quartile (Q3). A high average mapping quality was observed, with an average MAPQ value of ~69.1. The average insert sizes between corresponding read pairs ranged from 158 to 319 bp for the individual genotypes (Table 2 (available online only)). Regarding the proportion of the reference genome with sufficient read coverage, we observed large differences between genotypes particularly between natural versus synthetic accessions. This is expected due to large-scale chromosome rearrangements, including homoeologous non-reciprocal translocations, which are known to occur in synthetic *B. napus* during *de novo* allopolyploidisation [8]. Therefore we included only reference positions having a minimum of 4 reads aligned per reference position. The percentage of reference positions with sufficient read coverage (4) ranged between 51.3% (Q1) and 68.6% (Q3). Overall we discovered sufficient coverage for the natural *B. napus* accessions lines at 66.5% of all reference positions (Q1: 64.4% and Q3: 71.1%). In comparison, the synthetic *B. napus* accessions showed a significant overrepresentation of reference positions with missing data, with an average of only 48.4% (Q1: 41.1% and Q3: 57.5%) reference positions covered by at least 4 reads. Among the sequences with sufficient coverage in both groups, a higher sequence diversity compared to the *B. napus* Darmor-*bzh* reference sequence was observed in the synthetic accessions than in the adapted accessions.

To reveal INDEL mutations a gapped alignment is required that was constructed independently using *Bowtie2* (ref. 13). To achieve high confidence the respective alignments were performed using ‘−-very-sensitive’ setting plus usage of long seed length (‘−L 28’) and suppressing of unpaired alignment (--no-mixed’). We observed a 35% increase in the percentage of aligned read pairs when comparing the total number of properly paired reads between the un-gapped and the gapped read alignment. In comparison to the un-gapped read alignment a lower stringency was applied to tolerate gaps. As a consequence the number of sequence mismatches increased, however we observed good concordance when comparing the respective alignments. The resulting average insert size of corresponding read pairs, calculated for each genotype individually, proved to have only a slight difference of ~4 bp, whereby the percentage of observed difference in insert sizes ranged from 0.6% in the Q1 quartile to 2.4% in the Q3 quartile. Together with an identical global alignment quality (average MAPQ value >69) we conclude a high quality and a good overall alignment confidence. For visual inspection of the constructed alignments we used Tablet^14^, a graphical viewer for sequence assembly and alignment resources.

The *B. napus* Darmor-*bzh* v4.2 reference genome^1^ was constructed with extensive sequencing capacities using an estimated 21.2-fold coverage of the genome by 454 sequences. For subsequent scaffolding these were extended by multiple long distance mate pair sequence libraries (8 and 20 kb) and Sanger BES of an available BAC library. The construction of the final assembly involved careful investigation to fill the gaps between scaffolds and improve the sequences by error correction applied in the post-assembly phase. However, the final assembly still consists in 13.2% of the sequence base pairs of ‘N’s indicating an ambiguity for the respective nucleotides bases. The structure of the reference genome sequence is organized in 19 *B. napus* chromosomes. In addition, around 25% (204.9 Mbp) of the sequences of the Darmor-*bzh* v4.2 reference genome could not be assigned within the assembly process. These contigs were concatenated either in random chromosomes, where the contigs could be assigned to a chromosome, and in a random 'unknown' chromosome where no specific assignment was possible. Altogether, these reported characteristics of the reference genome indicate that the complexity of a genome and its repetitive regions even with today’s sequencing capacities is not an easy task, especially in an allopolyploid plant like *B. napus*.

In our read alignments (Table 2 (available online only)) both the natural and the adapted accessions show on average only a low proportion of reference bases covered by reads (on average 66.5% in the adapted and 48.4% in the synthetic lines for the stringent read alignment). One reason might be homopolymer repeat lengths errors in the *B. napus* Darmor-*bzh* v4.2 reference sequence, which appear extensively in data sequenced with the 454 technology^15^. This is consistent with the increased coverage rate monitored in the gapped alignment. Another possible cause may be the parameters used for the alignment. In this study we focused on the detection of high quality, position-specific SNPs. Therefore, we selected stringent options for the *SOAP2* alignment that exclude reads which are mapping with the same probability to more than one position within the genome. As the two subgenomes in the adapted accessions show a similarity of around 95%, a high proportion of reads was excluded from the alignment. The resynthesized lines include accessions with different parental origin. Due to this diversity we expect them to have only a low similarity with the reference genome of the adapted accession Darmor-*bzh*. Therefore stringent alignment parameters, allowing only two mismatches and no gaps, also lead to a high rate of unaligned reads, potentially leading to a low portion of reference bases being covered. This is also concordant with the results from the less stringent gapped alignment, showing a significantly higher rate of sequence coverage. Finally, for >13% the genomic sequence is not known and therefore is excluded from the read alignment process. In consequence, the genome coverage calculation relies on very stringent and cautious estimation settings and thus might be an underestimation. Relaxing the number of aligned reads (>=4), that are required to be considered as covered positions, affects this calculation substantially. Reducing this requirement to a single read the proportion of the genome coverage is substantially increased. On average 75.6% of the *B. napus* genome is covered for adapted and 65.1% for synthetic lines when analyzing the stringent *SOAP2* read alignment. This further is increased to 81.4% and 77.4, respectively, when the relaxed *Bowtie2* alignment is used. In summary, we are confident that the established read alignment resources are of high quality.

### SNP discovery

**Figure 1:**
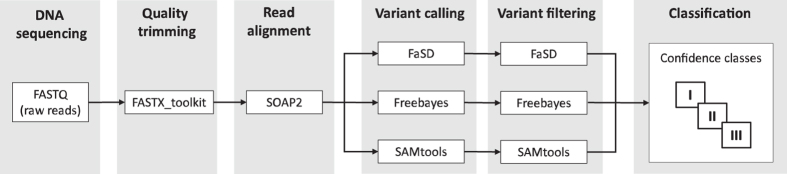
Schematic pipeline of the data processing procedures. The workflow is divided into six phases. Each genotype was processed individually by the pipeline to process the 685 Gbp of sequence data.

For the genome-wide discovery of variant positions (VP), a single sample SNP calling was utilized individually for each of the accessions. To reveal diversity with highest sensitivity we performed an approach using multiple variant-calling methods. Significant variability can be found in variant sites detected by different SNP calling methods^16^, making it advisable to apply multiple alternative tools to avoid overlooking informative sites. On the other hand, the low concordance of different variant-calling pipelines^17^ makes it necessary to carefully investigate variant positions. Hence we performed variant calling with three different prediction methods, using the tools *FaSD*^18^, *Freebayes*^19^ and *SAMtools*^12^, followed by analysis of the concordance of the calls during the variant filtering. The schematic workflow of the SNP discovery process is depicted in Fig. 1. For VP calling with *FaSD* we applied the parameter ‘−d 4’ and the two-score cutoff thresholds of ‘−c1 3.2’ and ‘−c2 15.8’. For VP calling with *Freebayes* we used the parameter settings ‘−C 4’, ‘−R 0’, ‘−m 20’ and ‘−F 0.002’. For VP calling using *SAMtools* we used the parameter settings ‘−q 20’ and ‘−Q 13’.

### Variant filtering

**Figure 2:**
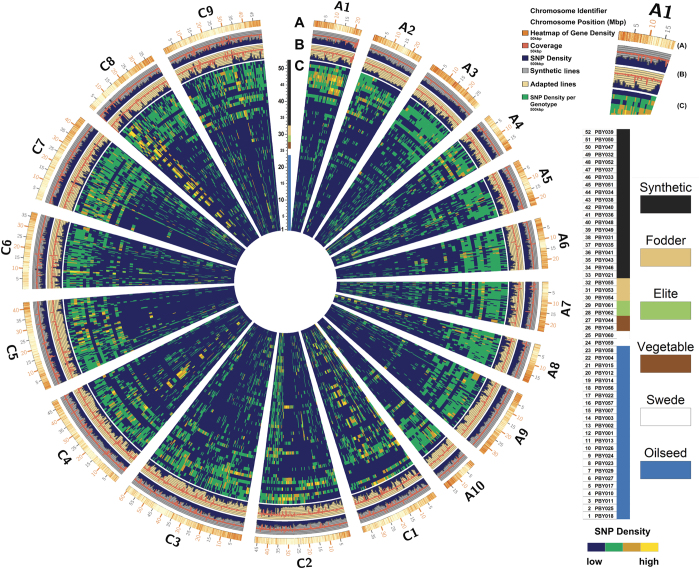
Diversity plot of 3.28 million variant positions (VPs, including SNPs and InDels) with defined chromosome positions visualized with *Circos*^20^. The illustration shows the 19 chromosomes of the *Brassica napus* genome, ten from the A subgenome and nine from the C subgenome. The outermost track (**A**) displays the position of genes and the corresponding scale in Mbp. The distribution of gene models shows that genes are abundant in distal euchromatin compared to the marginal packing in centromeric regions. The two subsequent tracks (**B**) illustrate the comparative mean diversity between the two subgroups of natural and synthetic *B. napus* accessions as a blue histogram plot, and the average read coverage as red line plot, respectively. The mean diversity is calculated for each of the subgroups by using the number of identified VPs in a 500 kb window, divided by the number of accessions per subgroup. The inner tracks (**C**) display the observed diversity (number of VPs) for each of the resequenced 52 genotypes, illustrated as heatmaps by using SNP densities in 500 kb windows. Genotypes are ordered by the six subgroups (synthetic, fodder, elite, vegetable, swede and oilseed) and within each subgroup by descending number of VPs.

A first posterior filtering was applied independently to all variant data sets (VCF files) before merging of the results from the multiple calling methods for construction of the final variant set. The independent application of multiple variant calling methods allowed us to use the variant caller count (VCC) as an additional confidence value for the prediction of variant positions. The VCC indicates how many variant calling methods predict a particular VP. All successful VP calls fulfil the following criteria in at least one of the genotypes: bi-allelic, SNP quality score >=100, homozygous, read depth >=4 and a VCC >=2. The final diversity set is published as a SNP matrix (Data Citation 4). To construct the SNP matrix the BAM files were converted into the PILEUP format using *SAMtools ‘mpileup’* and together with the discovered variant positions these information is integrated by a custom script (Data Citation 3). For each variant position and genotype the putative allele(s) for the different variants are shown. For differentiation of homozygous and heterozygous positions all alternative alleles are required to succeed the minimal read depth >=4. Genotypes with insufficient or no read information (read depth <4) are presented as ‘NA’, while positions with no alternative allele call are presented with the reference allele. A variant position is defined as homozygous when the allele frequency (AF) in one of the variants is <10% or >90%. Homozygous positions are displayed by the corresponding nucleotides (‘AA’, ‘CC’, ‘GG’, ‘TT’), heterozygous positions (10% <=AF <=90%) with both observed alleles. Per genotype we only present the bi-allelic relationship using the first two major alleles. For the minimum quality score per variant site a cut-off value of 100 was used. Due to the complex polyploid nature of the *B. napus* genome, heterozygous positions caused by mis-aligned reads from paralogues regions are common, hence we only report homozygous SNPs. The distribution of unique, genome-wide *B. napus* variant positions the 52 resequenced genomes were depicted graphically in a condensed circular layout constructed using the tool *Circos*^20^ (Fig. 2). For clarity of the layout all reference sequences without a defined chromosome position (‘random’) were discarded from the plot. The high number of unassigned contigs indicates putative assembly problems, which might affect the distribution of genomic variation. However, the read alignment and variant calling was performed on the complete reference genome to achieve a comprehensive representation of diversity within the studied genotypes.

### Repeat investigation

The *B. napus* Darmor-*bzh* genome assembly contains 34.5% transposable elements^1^. To gain knowledge about the repeat constitution in our genotype collection we performed a *k*-mer analysis, using an adapted *Kmasker*^21^ method focused on the repeat constitution of sequences surrounding the detected VP sites. For each of the 52 resequenced accessions we used ~1-fold sequence data (down sampling) to construct individual *k*-mer indices (totaling ~50-fold coverage). Each of these genotype-specific *k*-mer index was used to analyze the 100 bp flanking sequence (upstream and downstream) of a VP site. The combined and normalized results provided as data record represent a useful resource to estimate repeat occurrence (Data Citation 5). We found 545,846 VPs with increased *k*-mer values (>20) when calculating the average *k*-mer frequencies of flanking sequences. Within all these positions more than 40 genotypes (Q3) exhibited an increased *k*-mer frequency in either the left or the right flanking sequence. Validation in the *B. napus* Darmor-*bzh* reference genome demonstrated that, among the 4.3 million detected VPs, 693,004 (16.1%) were located in repeat regions and 3,610,807 (~83.9%) in non-repetitive reference positions. This analysis enables a more profound selection of VPs that are less likely to be affected by repeats.

### Discovery of insertion-deletion polymorphisms

**Figure 3:**
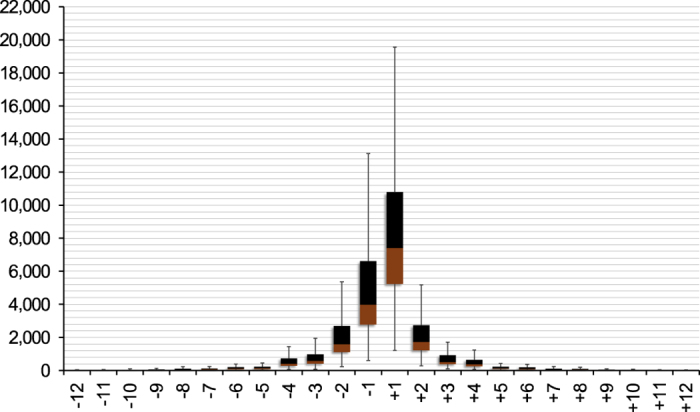
Distribution of discovered InDel sizes. InDel calls of all 52 genotypes were evaluated. The plot displays the range between the quartiles Q1 and Q3 for a particular InDel size. Around 50% of all genotypes had InDel numbers within the depicted range. The boundary between the lower (brown) and upper (black) field for a particular InDel length represents the average number of InDels of that length which were detected in all of the 52 sequenced accessions.

Calling of insertion-deletion polymorphisms (InDels) was performed independently from the SNP calling as a separate process, using a gapped alignment constructed with *Bowtie2*. BAM files of the read alignments were converted into PILEUP files using *SAMtools ‘mpileup’*. These were further processed by *BCFtools*^22^ to screen for insertion and deletions using the setting ‘−V indels’. Stringent parameter setting required a minimal base quality of ‘−Q 30’ and a minimal read alignment quality of ‘−q 20’. A posterior filtering was performed subsequently that is prioritizing InDel sites with a minimal (8) and maximal read depth (50), as well as a stringent IMF (0.9) and IDV (8) setting to identify high quality and homozygous sites. The latter two parameter control the maximal fraction of reads (IMF) and set the maximum number of reads (IDV) that support an InDel. In total we detected 633,844 insertions and 469,860 deletions in the range between −20 and 20 bp, whereby the large majority (~90%) are <=3 bp in length (Fig. 3). The highest number of InDels was detected in the synthetic *B. napus* accessions, where 9 out of 20 exceed the Q3 quartile (15,993 insertions and 11,852 deletions), whereas for the oilseed forms in only 3 of the 32 accessions an exaggerated InDel number was observed. On the other hand 13 accessions were detected to have fewer InDels than the Q1 quartile (7,313 insertions and 4,913 deletions), of which 10 are oilseed forms like the Darmor-*bzh* reference genotype. Accessions PBY047, which exhibited the highest number of insertions (28,972), and PBY0039, which exhibited the highest number of deletions (23,485), had more than 52.000 InDels, illustrating the high diversity within synthetic accessions. Analysis of InDels within coding regions of *B. napus* genes revealed a moderate enrichment of in-frame InDels (multiples of three nucleotides). This result is in accordance with previous findings in other species^23^.

### Functional annotation of variations

We investigated the functional effects of SNP and InDel variant positions (VPs), distinguishing between synonymous and non-synonymous variations using the tool *COOVAR*^24^. For SNPs the class of non-synonymous SNPs (nsSNPs) was further differentiated into ‘radical’ SNPs, which can cause loss of function (LoF), and ‘moderate’ SNPs, which have lower potential to affect the gene function. The third class ‘low’ includes synonymous SNPs (synSNPs). These variations locate in coding regions, but do not affect the protein sequence. The fourth class ‘other’ includes non-coding SNPs (ncSNPs) that locate in intronic or intergenic regions. As expected, the majority of detected SNPs belong to the latter two classes, with 85.2% classified as ‘other’ and 7.8% as ‘low’. The complete listing of the distribution of SNPs within these 4 classes is given in Table 3. A total of 67,892 SNPs were classified as LoF, hence these results provide a valuable resource to study functional sequence diversification in *B. napus*. In total 39,446 different genes are affected in at least one of the studied genotypes by at least one radical SNP leading to a predicted LoF. A total of 5,121 genes are modified by InDels that lead to a predicted LoF. For InDels again, the classes ‘LoF’ and ‘moderate’ have minor proportions, with 1.32% and 0.48% respectively. With 98.2% the large majority of InDels is observed in intronic or intergenic regions (‘other’). The full set of gained functional annotation of SNPs and InDels are provided as a GVF file for each genotype (Data Citation 6).

A considerable proportion of the 101k *B. napus* gene models showed disruptions of the coding sequence by mutations (LoF, moderate or low). In total, we observed 488 highly diverse genes, affected in more than 32 genotypes. Furthermore, 11,818 genes were disrupted in a medium number of genotypes (>5 and <=32) and 24,708 genes in a low numbers of genotypes (<5=). Interestingly, a total of 61,594 (61.0%) gene models showed little or no nucleotide variability.

### Code availability

All custom scripts applied for data processing are deposited as DOI (Data Citation 3). The developed custom scripts are available without restrictions. Applied software including version details are *BLASTN* (version 2.2.30), *SOAP2* (version 2.21), *Bowtie2* (version 2.2.5), *cutadapt* (version 1.8), *FastQC* (version 0.11.2), *FaSD* (version downloaded January 2015), *Freebayes* (version 0.9.21), *SAMtools* (version 0.1.19), *BCFtools* (version 1.2) and *e!DAL* (version 2.3.9). Applied parameter settings are described in the corresponding sections.

## Data Records

The SNP matrix of the 4.3 million variant positions is published as DOI (Data Citation 4)^4^. Functional annotation of SNPs and InDels are available as GVF files (Data Citation 6). Repeat investigations of sequences encapsulating the variant positions are available as DOI (Data Citation 5). Scripts applied for data processing are deposited as DOI (Data Citation 3). All DOIs were generated using the tool *e!DAL*^25^.

## Technical Validation

### Overlap with *Brassica napus* 60k genotyping data

As a measure of quality we compared the results of the VP calling method with the outcome from the Illumina 60k SNP Brassica Consortium Infinium genotyping array applied on the same samples. Flanking sequences from the SNP markers on the genotyping array were anchored to the *B. napus* Darmor-*bzh* reference sequence (v. 4.2), utilising a stringent *BLASTN* analysis^26,27^ with parameter ‘−perc_identity 98’, ‘−evalue 10’, ‘−word_size 11’. The majority of SNP markers are embedded in 201 bp (N50 length is 201 bp, with overall lengths ranging from 62 to 301 bp). This information was used to set anchoring criteria requiring that 90% of the marker sequence is linked with 98% identity to the reference sequence. From 52,157 functional, polymorphic markers called by the array in the 52 genotypes, we assigned 35,534 different positions for 32,333 unique, high-confidence markers. Among these, 23,871 diverse reference positions (corresponding to 73.7% or 23,841 SNP markers) showed a direct overlap to a detected VP in the 52 accessions. In addition, we utilized a less stringent anchoring, using only the 50 bp oligonucleotide sequences of the SNP markers, and found an additional set of 6,371 SNPs overlapping the VPs that were identified in our study. In total, 30,242 diverse reference positions (23,871 with high stringency and 6,371 with relaxed stringency) could be directly validated by a marker from the 60k SNP array.

To verify the correctness of variant calling we further validated the concordance of alleles at detected positions with the allele information of the genotyping assay. In 99.87% of the stringently assigned SNP markers (99.08% in the relaxed assignment) we found concordance between the two data sets, except for cases where missing marker calls from the Infinium assay can be attributed to excessively stringent filtering of SNPs in the variant calling process. Non-concordance can also result from incomplete anchoring of markers to the reference sequence. Nevertheless, with over 30,000 validated marker positions and a high allele concordance of over 99.76%, we conclude an exceptionally high quality of the SNP predictions.

## Usage Notes

Access to the plant materials described in this paper is possible for research purposes via a material transfer agreement with the material owners. Please contact Dr. Gunhild Leckband (g.leckband@german-seed-alliance.de).

## Additional Information

Table 2 is only available in the online version of this paper.

**How to cite this article:** Schmutzer, T. *et al.* Species-wide genome sequence and nucleotide polymorphisms from the model allopolyploid plant *Brassica napus*. *Sci. Data* 2:150072 doi: 10.1038/sdata.2015.72 (2015).

## Supplementary Material



## Figures and Tables

**Table 1:** Origins of 20 synthetic accessions

**Accession name**	**Genotype Code**	**Form**	**Mother**	**Father**
**Resyn-Go S4**	PBY021	Synthetic	*B. rapa* ssp.*rapa*	*B.oleracea* convar. *acephala*
**G 50**	PBY031	Synthetic	*B. oleracea* conv. *acephala* var. *gongyloides*	*B. rapa* ssp. *oleifera*
**H44**	PBY032	Synthetic	*B. oleracea* conv. *capitata* var. *sabauda*	*B. rapa* ssp. *pekinensis*
**H 149**	PBY033	Synthetic	*B. oleracea* conv. *capitata* var. *medullosa*	*B. rapa* ssp. *chinensis*
**H 165**	PBY034	Synthetic	*B. oleracea* conv. *capitata* var. *sabauda*	*B. rapa* ssp. *chinensis*
**H 176**	PBY035	Synthetic	*B. oleracea* conv. *capitata* var. *sabauda*	*B. rapa* ssp. *pekinensis* var. l*axa*
**R53**	PBY036	Synthetic	*B. oleracea* conv. *capitata* var. *sabellica*	*B. rapa* ssp. *pekinensis*
**R76**	PBY037	Synthetic	B. *oleracea* conv. *botrytis* var. *alboglabra*	*B. rapa* ssp. *oleifera*
**RS 4/6**	PBY038	Synthetic	*B. rapa* ssp. *pekinensis*	*B. oler.*conv. *capitata* var. *sabellica* x *B. oler.* conv. *gemmifera*
**RS 10/7**	PBY039	Synthetic	*B. rapa* ssp. *trilocularis*	*B. oleracea* conv. *fructicosa*
**RS 13/6**	PBY040	Synthetic	*B. rapa* ssp. *pekinensis*	*B. oleracea* conv. *botrytis* var. *alboglabra*
**S 13**	PBY041	Synthetic	*B. rapa* ssp. *oleifera* 4x	*B. oleracea* conv. *capitata* var. *medullosa*
**OLL 1**	PBY043	Synthetic	*B. rapa* ssp. *oleifera*	B. *oleracea* ssp. o*leracea*
**R 99**	PBY046	Synthetic	*B. oleracea* conv. *capitata* var. *capitata*	*B. rapa* ssp. *pekinensis*
**RS 7/6**	PBY047	Synthetic	*B. rapa* ssp. *pekinensis*	*B. oleracea* conv. *capitata* var. *medullosa*
**RS 8/6**	PBY048	Synthetic	*B. rapa* ssp. *pekinensis*	*B. oler.* conv. *capitata* var. *medullosa* x *B. oler.* conv. *gemmifera*
**S 39**	PBY049	Synthetic	*B. oleracea* conv. *capitata* var. *capitata*	*B. rapa* ssp. *chinensis*
**CRY 1**	PBY050	Synthetic	*B. rapa* ssp. *trilocularis*	*B. cretica*
**HIY 1**	PBY051	Synthetic	*B. rapa* ssp. *trilocularis*	*B. hilarionis*
**MOY 4**	PBY052	Synthetic	*B. rapa* ssp. *trilocularis*	*B. montana*
For each of the synthetic forms in the PBY collection the parents are given to illustrate the origin.				

**Table 2:** Summary of re-sequencing data and read alignment metrics

**Genotype information**			**Raw reads**			**Quality- trimmed reads**			**Un-gapped read alignment (SNPs)**							**Gapped read alignment (InDels)**						
**Accession name**	**Genotype Code**	**Form**	**Paired-end reads**	**GC percent**	**X-fold coverage**	**Paired end reads (quality trimmed)**	**GC content**	**X-fold coverage**	**Number of aligned and paired reads**	**Properly paired in alignment [%]**	**Average quality**	**Insert size average (bp)**	**Insert size standard deviation (bp)**	**Reference genome covered by >4 reads (bp)**	**Percentage of reference genome covered by >4 reads**	**Number of aligned and paired reads**	**Properly paired in alignment [%]**	**Average quality**	**Insert size average (bp)**	**Insert size standard deviation (bp)**	**Reference genome covered by >4 reads (bp)**	**Percentage of reference genome covered by >4 reads**
**Dippes**	PBY001	Oilseed	76,678,771	36.07	13.57	59,784,163	35.15	10.55	44,694,294	74.76	69.2	190	43.3	574,275,985	67.54	56,268,693	94.12	69.2	192	44.70	621,867,140	73.14
**Gross-Luesewitzer**	PBY002	Oilseed	94,147,683	36.44	16.66	70,776,879	35.37	12.49	51,737,830	73.10	69.1	180	42.3	589,786,241	69.36	66,533,247	94.00	69.2	181	43.80	637,384,608	74.96
**Jupiter**	PBY003	Oilseed	82,683,075	36.25	14.63	60,770,841	35.61	10.72	42,836,550	70.49	69.1	289	93.5	558,142,391	65.64	53,431,420	87.92	69.1	278	85.90	613,742,287	72.18
**Kromerska**	PBY004	Oilseed	221,452,664	35.98	39.20	156,338,699	35.07	27.59	105,796,574	67.67	69.0	298	97.7	652,752,882	76.77	137,254,345	87.79	69.0	287	90.60	690,951,227	81.26
**Major**	PBY007	Oilseed	86,415,347	36.36	15.29	65,912,007	35.35	11.63	48,544,782	73.65	69.1	194	50.4	573,829,678	67.49	61,525,463	93.34	69.2	195	51.30	622,722,929	73.24
**Skziverskij**	PBY010	Oilseed	43,855,559	36.66	7.76	39,006,814	36.33	6.89	29,448,099	75.49	69.4	208	51.7	426,946,858	50.21	36,559,645	93.73	69.4	209	52.70	486,974,475	57.27
**Start**	PBY011	Oilseed	48,236,482	36.55	8.54	43,135,096	36.26	7.62	33,180,006	76.92	69.4	214	52.0	493,108,977	57.99	40,646,849	94.23	69.4	214	52.80	541,202,802	63.65
**Aragon**	PBY012	Oilseed	116,546,982	36.26	20.63	81,161,229	34.92	14.32	59,827,873	73.71	69.0	200	50.4	612,921,838	72.08	76,074,196	93.73	69.0	201	51.60	655,908,047	77.14
**Beluga**	PBY013	Oilseed	88,007,335	36.35	15.58	68,115,427	35.41	12.02	51,144,032	75.08	69.2	192	47.8	603,238,432	70.94	64,012,428	93.98	69.2	193	48.90	643,354,941	75.66
**Olimpiade**	PBY014	Oilseed	75,136,422	36.71	13.30	56,571,878	35.34	9.98	41,071,490	72.60	69.0	171	42.0	535,429,640	62.97	52,992,427	93.67	69.0	172	43.40	593,896,859	69.85
**Wotan**	PBY015	Oilseed	99,373,470	36.30	17.59	75,603,290	35.58	13.35	55,729,238	73.71	69.1	202	48.4	606,916,516	71.38	70,982,677	93.89	69.1	203	49.60	653,524,802	76.86
**Canberra x Courage DH**	PBY017	Oilseed	81,363,434	36.24	14.40	59,434,864	35.62	10.48	44,036,290	74.09	69.1	301	86.3	566,104,615	66.58	53,994,885	90.85	69.1	293	83.60	611,690,770	71.94
**Darmor**	PBY018	Oilseed	91,568,141	36.35	16.21	66,926,680	35.18	11.81	52,712,525	78.76	69.1	183	44.2	645,346,272	75.90	63,758,847	95.27	69.1	185	45.90	662,009,080	77.86
**Resyn-Go S4**	PBY021	Synthetic	58,724,730	36.41	10.39	44,243,684	35.81	7.81	32,413,472	73.26	69.1	227	82.3	476,529,225	56.04	40,339,341	91.18	69.1	224	77.40	535,689,158	63.00
**Alaska**	PBY022	Oilseed	109,827,023	36.31	19.44	75,705,714	34.95	13.36	55,523,963	73.34	69.0	196	51.8	611,910,833	71.96	70,658,742	93.33	69.0	197	53.10	652,374,189	76.72
**Pirola**	PBY023	Oilseed	66,697,170	36.67	11.80	48,166,363	35.22	8.49	35,879,099	74.49	69.0	160	38.4	514,381,538	60.49	45,025,535	93.48	69.0	161	40.60	562,153,242	66.11
**Rapid**	PBY024	Oilseed	87,861,325	36.31	15.55	61,778,308	34.89	10.89	45,304,316	73.33	68.9	207	51.1	554,202,556	65.18	58,094,485	94.04	69.0	208	51.90	608,752,906	71.59
**Pacific**	PBY025	Oilseed	60,057,624	36.09	10.63	43,187,827	35.44	7.62	31,745,436	73.50	69.1	311	92.7	461,996,374	54.33	38,168,913	88.38	69.1	300	86.90	511,940,298	60.21
**Savannah**	PBY026	Oilseed	89,632,389	36.21	15.86	61,795,222	35.38	10.90	44,445,830	71.92	69.0	312	87.2	564,166,288	66.35	55,541,939	89.88	69.0	304	85.10	618,531,215	72.74
**Vivol**	PBY027	Oilseed	84,211,160	36.18	14.90	57,826,789	35.28	10.20	41,981,621	72.60	69.0	319	83.1	567,378,895	66.73	51,951,765	89.84	69.0	310	81.70	617,267,311	72.59
**Expert**	PBY029	Oilseed	90,794,664	36.19	16.07	63,499,906	34.94	11.20	47,915,177	75.45	69.0	200	49.7	588,869,405	69.25	59,906,715	94.34	69.0	201	50.90	628,916,009	73.96
**G 50**	PBY031	Synthetic	69,292,095	36.45	12.26	52,124,064	35.41	9.20	36,045,422	69.15	69.1	178	41.1	487,156,619	57.29	48,497,178	93.04	69.1	180	43.10	554,066,351	65.16
**H44**	PBY032	Synthetic	81,342,525	36.05	14.40	58,060,866	35.24	10.24	31,380,639	54.05	69.1	303	80.6	352,818,694	41.49	49,716,664	85.63	69.1	296	79.20	505,875,655	59.49
**H 149**	PBY033	Synthetic	119,715,932	36.84	21.19	87,460,199	35.41	15.41	56,753,965	64.89	69.0	180	44.3	518,733,148	61.01	80,844,771	92.44	69.0	182	45.90	596,578,095	70.16
**H 165**	PBY034	Synthetic	84,221,458	36.51	14.91	65,696,304	35.42	11.58	41,587,341	63.30	69.1	172	38.6	437,676,631	51.47	60,464,043	92.04	69.1	173	40.40	532,940,940	62.68
**H 176**	PBY035	Synthetic	61,418,643	36.55	10.87	45,834,995	35.30	8.08	31,463,046	68.64	69.0	158	36.7	445,138,849	52.35	42,526,409	92.78	69.0	160	39.30	513,594,698	60.40
**R53**	PBY036	Synthetic	83,546,570	36.15	14.79	60,205,621	35.37	10.62	37,056,142	61.55	69.1	296	79.2	430,083,214	50.58	53,441,660	88.77	69.1	290	77.90	540,628,366	63.58
**R76**	PBY037	Synthetic	98,794,991	35.98	17.49	64,143,850	35.04	11.31	35,388,261	55.17	69.0	317	91.8	359,020,986	42.22	53,433,110	83.30	69.0	305	85.40	506,095,979	59.52
**RS 4/6**	PBY038	Synthetic	81,483,259	36.08	14.42	54,498,882	35.20	9.62	31,029,708	56.94	69.0	304	92.1	336,708,852	39.60	46,201,391	84.77	69.0	295	86.30	479,768,131	56.42
**RS 10/7**	PBY039	Synthetic	94,857,523	36.48	16.79	67,633,538	35.06	11.91	38,084,714	56.31	68.9	204	48.7	339,969,197	39.98	61,088,658	90.32	69.0	205	49.00	493,189,002	58.00
**RS 13/6**	PBY040	Synthetic	82,610,127	36.24	14.62	55,260,011	35.31	9.75	29,984,948	54.26	69.0	293	89.3	368,149,785	43.30	46,680,728	84.47	69.0	284	83.80	530,955,765	62.44
**S 13**	PBY041	Synthetic	70,965,496	36.39	12.56	59,586,472	35.91	10.53	44,004,589	73.85	69.2	212	50.7	562,282,029	66.13	55,810,435	93.66	69.3	213	51.40	617,298,198	72.60
**OLL 1**	PBY043	Synthetic	126,776,353	36.55	22.44	91,718,467	35.20	16.17	55,323,797	60.32	69.0	169	39.5	557,150,401	65.52	84,932,745	92.60	69.0	169	40.30	631,219,134	74.24
**Chuosenshu**	PBY044	Vegetable	100,155,618	36.04	17.73	73,046,310	34.90	12.89	53,417,179	73.12	69.1	191	48.3	582,060,841	68.45	68,384,909	93.62	69.1	193	49.70	633,249,610	74.47
**Grüner Schnittkohl**	PBY045	Vegetable	71,064,314	36.85	12.58	53,814,404	35.51	9.48	39,276,697	72.98	69.1	185	43.4	504,681,041	59.35	50,391,710	93.64	69.1	186	45.00	567,794,080	66.78
**R 99**	PBY046	Synthetic	50,101,606	36.41	8.87	39,869,551	35.43	7.03	27,671,055	69.40	69.1	158	41.1	378,685,300	44.54	36,736,402	92.14	69.1	159	41.80	441,672,434	51.94
**RS 7/6**	PBY047	Synthetic	89,334,621	36.46	15.81	63,532,993	34.96	11.19	32,635,263	51.36	69.0	199	46.3	376,174,589	44.24	57,141,393	89.94	69.0	200	47.70	541,252,777	63.65
**RS 8/6**	PBY048	Synthetic	112,253,074	36.39	19.87	79,432,078	34.97	14.00	44,616,185	56.16	69.0	197	46.3	495,240,889	58.24	73,354,735	92.35	69.0	198	47.30	605,072,064	71.16
**S 39**	PBY049	Synthetic	94,470,389	36.16	16.72	71,720,158	35.46	12.67	48,963,355	68.27	69.1	209	50.6	540,592,853	63.58	66,876,537	93.25	69.1	209	51.20	606,649,247	71.35
**CRY 1**	PBY050	Synthetic	90,794,664	36.19	16.07	61,950,538	35.36	10.93	30,156,437	48.68	69.0	307	88.5	252,335,100	29.68	51,093,132	82.47	69.0	298	83.50	466,891,231	54.91
**HIY 1**	PBY051	Synthetic	78,128,948	36.35	13.83	54,999,607	35.56	9.70	29,920,622	54.40	69.0	306	89.1	311,071,983	36.58	46,187,021	83.98	69.1	297	84.40	482,891,235	56.79
**MOY 4**	PBY052	Synthetic	79,060,123	36.38	13.99	55,935,096	35.60	9.87	25,379,215	45.37	69.0	306	84.7	210,927,095	24.81	45,743,860	81.78	69.1	297	81.40	445,926,978	52.44
**English Giant 194**	PBY053	Fodder	83,612,186	36.06	14.80	61,894,909	35.23	10.92	43,333,520	70.01	69.1	303	93.8	550,031,128	64.69	53,944,017	87.15	69.1	292	87.70	610,712,975	71.82
**Nunsdale**	PBY054	Fodder	91,414,162	36.67	16.18	69,396,326	35.42	12.24	50,155,140	72.27	69.0	179	42.3	570,416,274	67.08	65,012,943	93.68	69.0	180	44.10	625,170,716	73.52
**Palu**	PBY055	Fodder	100,184,910	36.60	17.73	79,508,952	35.56	14.02	56,721,442	71.34	69.1	161	38.7	590,851,827	69.49	74,144,376	93.25	69.1	162	40.20	643,983,682	75.74
**Abukuma Natane**	PBY056	Oilseed	98,085,738	36.22	17.36	67,332,540	34.87	11.88	49,633,312	73.71	69.0	204	47.3	589,653,819	69.35	63,121,031	93.75	69.0	205	48.80	634,545,437	74.63
**E94197**	PBY057	Oilseed	111,406,405	36.25	19.72	80,774,762	35.07	14.25	60,211,568	74.54	69.1	199	48.0	620,305,628	72.95	76,312,738	94.48	69.1	200	49.00	658,435,307	77.44
**Evvin**	PBY058	Oilseed	92,253,176	36.41	16.33	71,127,080	35.43	12.55	49,724,957	69.91	69.2	199	47.8	540,442,856	63.56	66,073,934	92.90	69.2	200	48.80	617,691,761	72.64
**Fortin Family**	PBY059	Swede	80,681,462	36.26	14.28	58,661,174	35.09	10.35	43,027,470	73.35	69.1	198	47.2	577,626,135	67.93	55,233,597	94.16	69.1	200	48.40	621,256,303	73.06
**Sensation NZ**	PBY060	Swede	78,213,230	36.36	13.84	65,223,432	35.84	11.52	28,683,721	43.98	69.2	209	53.0	392,095,197	46.11	60,543,301	92.82	69.3	211	53.70	590,944,858	69.50
DH 5 (Oase*Nugget)	PBY061	Elite	148,656,552	36.52	26.31	123,516,260	35.98	21.82	90,648,622	73.39	69.2	204	50.1	658,910,322	77.49	116,942,074	94.68	69.2	205	50.40	689,284,408	81.06
**MSL007C**	PBY062	Elite	115,141,387	36.03	20.38	87,228,004	35.23	15.40	60,380,183	69.22	69.1	292	81.4	621,299,530	73.07	79,299,560	90.91	69.1	286	79.20	664,007,118	78.09
**Total**			4,673,308,987	1,889.61	827.13	3,440,929,123	1,838.34	607.06	2,328,627,012	3,517.77	3,591.9	11,743	3,106.8	26,336,526,251	3,097.31	3,143,897,619	4,745.76	3,592.7	11,637	3,076.70	30,420,496,830	3,577.61
**Average**			89,871,327	36.34	15.91	66,171,714	35.35	11.67	44,781,289	67.65	69.1	226	59.7	506,471,659	59.56	60,459,570	91.26	69.1	224	59.17	585,009,554	68.80
**Minimum**			43,855,559	35.98	7.76	39,006,814	34.87	6.89	25,379,215	43.98	68.9	158	36.7	210,927,095	24.81	36,559,645	81.78	69.0	159	39.30	441,672,434	51.94
**Maximum**			221,452,664	36.85	39.20	156,338,699	36.33	27.59	105,796,574	78.76	69.4	319	97.7	658,910,322	77.49	137,254,345	95.27	69.4	310	90.60	690,951,227	81.26
**Q1 quartile**			78,192,160	36.19	13.84	56,412,683	35.13	9.95	33,043,820	62.86	69.0	189	44.3	435,778,277	51.25	50,222,949	89.87	69.0	190	45.90	535,002,104	62.92
**Q3 quartile**			98,263,051	36.46	17.39	71,275,350	35.47	12.58	51,292,482	73.66	69.1	294	82.5	583,762,982	68.65	66,619,070	93.73	69.1	286	79.75	631,726,753	74.30
Detailed description of the whole-genome re-sequencing data from 52 *Brassica napus* accessions and corresponding read alignment performance.																						

**Table 3:** Functional annotation of SNPs and InDels

**Variant type**	**Minor allele frequency**	**LoF (frame shift InDel, splice acceptor or donor, stop gained or lost, non-conservative)**	**Moderate (inframe InDel, conservative missense)**	**Low (synonymous)**	**Other (intronic or intergenic)**	**Total**
SNP	>=0.5%	865 (1.46%)	2,802 (4.74%)	3,549 (6.00%)	51,891 (87.79%)	59,107
	0.1–0.5%	12,115 (1.64%)	42,667 (5.79%)	59,778 (8.11%)	622,693 (84.46%)	737,253
	<0.1%	54,912 (1.57%)	186,261 (5.31%)	272,773 (7.77%)	2,994,658 (85.35%)	3,508,604
	all	67,892 (1.58%)	231,730 (5.38%)	336,100 (7.81%)	3,669,242 (85.23%)	4,304,964
InDel	all	14,051 (1.32%)	5,083 (0.48%)	—	1,043,726 (98.20%)	1,062,860
The functional annotation of detected variants is summarised for different minor allele frequencies (MAF) and is categorized in 4 impact groups: 1) Loss of function (LoF), 2) moderate, 3) low and 4) other. An explanation of each group is provided in the text. Displayed percentages illustrate the proportion of each impact group within a MAF sub-category (row).						
